# Post-Infection Oscillometry and Pulmonary Metrics in SARS-CoV-2 Patients: A 40-Day Follow-Up Study

**DOI:** 10.3390/diseases11030102

**Published:** 2023-08-05

**Authors:** Noemi Suppini, Cristian Oancea, Ovidiu Fira-Mladinescu, Daniel Traila, Camelia Pescaru, Monica Steluta Marc, Diana Manolescu, Emanuela Vastag, Ayesha Ali, Elena Hogea, Ciprian Nicolae Pilut

**Affiliations:** 1 Discipline of Pulmonology, “Victor Babes” University of Medicine and Pharmacy Timisoara, 300041 Timisoara, Romania; noemi.suppini@umft.ro (N.S.); oancea@umft.ro (C.O.); mladinescu@umft.ro (O.F.-M.); traila.daniel@umft.ro (D.T.); pescaru.camelia@umft.ro (C.P.); marc.monica@umft.ro (M.S.M.); emanuela.tudorache@umft.ro (E.V.); 2 Center for Research and Innovation in Precision Medicine of Respiratory Diseases (CRIPMRD), “Victor Babes” University of Medicine and Pharmacy Timisoara, 300041 Timisoara, Romania; dmanolescu@umft.ro; 3 Doctoral School, “Victor Babes” University of Medicine and Pharmacy Timisoara, 300041 Timisoara, Romania; 4 Department of Radiology and Medical Imaging, “Victor Babes” University of Medicine and Pharmacy Timisoara, 300041 Timisoara, Romania; 5 Bhaskar Medical College, Amdapur Road 156-162, Hyderabad 500075, Telangana State, India; ayeshaaliflossy@gmail.com; 6 Department of Microbiology, “Victor Babes” University of Medicine and Pharmacy Timisoara, 300041 Timisoara, Romania; pilut.ciprian@umft.ro

**Keywords:** COVID-19, SARS-CoV-2, pulmonary function tests, oscillometry

## Abstract

The COVID-19 pandemic, caused by the SARS-CoV-2 virus, has had significant impacts on pulmonary function. This study aimed to comprehensively evaluate pulmonary function and structure in patients 40 days post-SARS-CoV-2 infection, employing an array of testing methodologies including spirometry, plethysmography, forced oscillometry, and CT scanning. It also sought to establish potential correlations between these metrics and evaluate if forced oscillometry could provide additional value in post-infective lung function assessment. A 40-day post-infection follow-up observational study was conducted involving 66 patients with confirmed SARS-CoV-2 infection. The results revealed decreases in FVC and FEF25–75 with the increasing severity of COVID-19. Specifically, patients with severe symptoms exhibited statistically significant decreases in FVC (mean = 86.8) compared with those with mild symptoms (mean = 106.0; *p* = 0.018). The FEF25–75 showed a similar trend, with severe patients exhibiting a mean of 77.7 compared with 82.9 in the mild group (*p* = 0.017). Furthermore, resonant frequency (RF) increased with disease severity, with the severe group exhibiting a statistically significant increase (mean = 17.4) compared with the mild group (mean = 14.3; *p* = 0.042). CT scans showed an increase in ground-glass opacities with disease severity, with 81.8% of severe patients demonstrating this finding (*p* = 0.037). Multiple regression analysis revealed that Reactance at 4 Hz (X4), Forced Expiratory Flow 25–75% (FEF25–75), and Resonant Frequency (RF) were significantly related to COVID-19 severity. Specifically, for each unit increase in these factors, the risk of the event was estimated to increase by a factor of 3.16, 2.09, and 1.90, respectively. Conversely, Resistance at 4 Hz (R4) and Airway Resistance (RAW) were found to significantly decrease the event hazard, highlighting their potential protective role. Spirometry, plethysmography, and forced oscillometry are effective in assessing these changes. Forced oscillometry may be particularly beneficial in identifying subtle changes in lung function post-COVID-19. Further studies are warranted to validate these findings and develop strategies to manage post-infective pulmonary changes in SARS-CoV-2 patients.

## 1. Introduction

Severe acute respiratory syndrome coronavirus 2 (SARS-CoV-2), responsible for the COVID-19 pandemic, has dramatically impacted global health and continues to be the subject of intensive medical research [1,2]. While the acute respiratory effects of SARS-CoV-2 infection are well-documented, there is an increasing concern regarding the potential long-term pulmonary complications in recovered patients [3,4]. In this context, the comprehensive evaluation of pulmonary function and structure post-infection becomes crucial for monitoring disease progression and optimizing patient care.

Pulmonary function tests (PFTs), such as spirometry and body plethysmography, along with chest computed tomography (CT), have traditionally been used to assess lung capacity and to detect and quantify lung abnormalities [5,6,7,8]. Spirometry allows for the measurement of dynamic lung volumes, whilst body plethysmography assesses static lung volumes, contributing to an overall and complete evaluation of pulmonary function [9]. CT, on the other hand, offers a detailed visualization of lung composition and can help identify specific structural changes post-infection [10].

Nevertheless, these traditional methods, although informative, might not capture all the functional and structural alterations post-SARS-CoV-2 infection, such as small airway disfunction, which was more evident in some studies when using oscillometry methods [11,12]. Forced oscillometry, a noninvasive technique for measuring respiratory impedance, provides additional parameters of respiratory mechanics not typically obtained from conventional spirometry or body plethysmography. By supplying information about the respiratory system’s resistance and reactance, forced oscillometry could add valuable insight into post-infective lung function alterations, potentially helping to detect and monitor subtler changes that might be overlooked by traditional assessments [13].

The importance of combined approaches in respiratory evaluation has been increasingly recognized in the literature. For example, the integration of spirometry, body plethysmography, and forced oscillometry has been shown to provide a more holistic view of respiratory status in conditions such as asthma and chronic obstructive pulmonary disease (COPD) [14]. In the context of SARS-CoV-2, where the long-term impacts on lung function and structure are still being elucidated, such an integrative approach might be particularly relevant.

Therefore, the present study aims to provide a comprehensive evaluation of pulmonary function and structure in patients with acute SARS-CoV-2 infection, at 40 days post-infection. This time frame was chosen as it represents a critical window for observing initial recovery and potential complications related to the infection, allowing for an analysis of immediate physiological responses without delving into the complexities of long-term post-infective syndromes such as long COVID. The primary objectives of this study were to evaluate the utility and feasibility of integrating spirometric parameters, plethysmography, forced oscillometry, and CT in assessing lung function and composition post-SARS-CoV-2 infection, and to elucidate potential correlations between these parameters. Additionally, we aimed to determine if forced oscillometry could add value to the conventional evaluation of post-infective lung function by identifying subtler changes potentially missed when using spirometry and body plethysmography.

## 2. Materials and Methods

### 2.1. Design and Ethics

An observational study was conducted to evaluate the pulmonary function in 66 patients after SARS-CoV-2 infection, assessed at 40 days post-infection. All patients were discharged from the “Victor Babes” Hospital for Infectious Diseases and Pulmonology in Timisoara, Romania. This study was conducted according to the guidelines of the Declaration of Helsinki and given the approval number 1136. 

### 2.2. Inclusion and Exclusion Criteria

The inclusion criteria encompassed individuals aged 18 years or above at the time of recruitment, having had a confirmed infection with the SARS-CoV-2 virus, which must have been established through a positive RT-PCR or antigen test. These individuals must have been clinically stable and physically capable of performing the necessary tests without discomfort or risk. Only those who agreed to provide informed consent were included in the study. Exclusion criteria included individuals who did not have a confirmed infection with SARS-CoV-2, those incapable of performing the necessary tests, and those with neurological, psychiatric, or unstable medical conditions that could interfere with the testing process or interpretation of results. Individuals with pre-existing pulmonary diseases were also excluded to prevent potential confounding of the pulmonary function results.

### 2.3. Definitions and Methods

In the present study, the following variables were meticulously considered for analysis: We classified the severity of SARS-CoV-2 infection and recorded the period of testing post-infection. Demographic data collected included personal numeric code (unique identifying number), age, sex, height, weight, and location of residence (either urban or rural). We took into consideration comorbidities and smoking status of the participants. Lung function was evaluated through multiple modalities. Spirometry was employed to measure Forced Vital Capacity (FVC), Forced Expiratory Volume in one second (FEV1), Airway Microvascular Permeability (AMPI), and Forced Expiratory Flow at 25–75% (FEF25–75). Ventilatory dysfunction was identified and noted.

Plethysmography tests were utilized to determine Total Lung Capacity (TLC), Residual Volume (RV), Specific Airway Resistance (sRAW), Airway Resistance (RAW), Airway Conductance (GAW), and the ratio and percentage of Residual Volume to Total Lung Capacity (RV/TLC and RV/TLC%). Interpretations of plethysmography results were recorded. Oscillometry is a method used to evaluate the mechanical properties of the respiratory system. Unlike traditional pulmonary function tests, it does not require forceful breathing maneuvers and is, therefore, more suitable for certain populations, including children or individuals with severe respiratory disease. In our study, several oscillometry parameters were measured, including Resonant Frequency (RF), Resistance at 4 Hz (R4), 6 Hz (R6), and 20 Hz (R20), and Reactance at 4 Hz (X4) and 6 Hz (X6). Resistance at different frequencies (R4, R6, R20) provides information about the obstruction or constriction in the airways. Higher resistance values usually indicate an increased difficulty in airflow, commonly found in conditions such as asthma or COPD. RF measures the point where resistance and reactance are equal. Changes in RF can indicate alterations in airway geometry, helping to understand diseases affecting airway flexibility. Reactance at 4 Hz and 6 Hz provides information about the elastic properties of the lung. It may offer insight into lung stiffness or compliance, helping in the diagnosis of conditions like fibrosis. 

Additionally, computerized tomography (CT) scans were carried out to assess lung composition and pathology. These analyses allowed for the determination of total lung capacity as well as the capacity of the right and left lungs separately. The scans helped identify the presence and volume of emphysema, normal lung tissue, ground-glass opacities, crazy-paving patterns, and lung consolidations. CT scans also facilitated the visualization and recording of calcifications and blood vessels. The computed tomography score was established for each patient. 

The Cosmed Quark PFT equipment was used to determine the pulmonary function tests. Spirometry, body plethysmography, and forced oscillometry were performed using appropriate equipment, in accordance with standard guidelines. In addition, a CT was performed to evaluate lung structure and capacity, aided by software for a thorough analysis of lung composition. All pulmonary tests were performed based on the American Thoracic Society and the European Respiratory Society (ATS-ERS) guidelines [15].

A total of 66 unvaccinated patients were selected and stratified by COVID-19 severity into three equal study groups, matched by age, gender, body mass index, and smoking status. Unvaccinated patients were selected to avoid the confounding effect that vaccination might have on disease severity and outcomes. During the recruitment phase, the SARS-CoV-2 Omicron variant was predominant [16]. The facemasks used by the researchers and the patients in the study were FFP2, with a minimum of 94% filtration percentage. The classification of the severity of patients’ COVID-19 infection was conducted according to World Health Organization (WHO) guidelines [17]. In our study, we categorized patients into mild, moderate, and severe categories based on symptoms, clinical signs, and oxygen saturation levels. Mild COVID-19 patients exhibited common respiratory symptoms without any signs of severe pneumonia or decreased oxygen saturation levels (above 94%). Moderate COVID-19 patients showed signs of pneumonia on chest imaging and had oxygen saturation levels between 91% and 94% but did not exhibit severe respiratory distress. Severe COVID-19 patients exhibited life-threatening symptoms such as acute respiratory distress syndrome (ARDS), with oxygen saturation levels below 90%, and potentially required intensive care or mechanical ventilation.

### 2.4. Statistical Analysis

Continuous data are presented as means with standard deviations (SD), while categorical data are summarized as frequencies and percentages. Differences between groups for continuous data were tested using Student’s *t*-test or ANOVA, and the Mann–Whitney U-test or the Kruskal–Wallis test for non-parametric data. Categorical data differences were tested using the χ^2^ test or Fisher’s exact test when expected cell counts were less than five. A cox regression model was used for the analysis of prognostic factors for COVID-19 severity based on lung parameters. All statistical analyses were performed using SPSS v.26 (IBM Corporation, Armonk, NY, USA).

## 3. Results

### 3.1. Patients’ Background

The study consisted of a cohort of 66 participants. The participants were evenly distributed into three severity groups: mild, moderate, and severe, each consisting of 22 individuals. The average age of the participants in the mild group was 54.1 ± 9.8 years, with the moderate and severe groups demonstrating slightly higher averages of 57.2 ± 8.6 years and 58.3 ± 11.0 years, respectively. Despite the trend towards higher average ages with increasing severities of illness, the observed differences did not reach statistical significance (*p*-value = 0.347). Body Mass Indexes (BMI) were also similar across groups. Participants in the mild, moderate, and severe groups had mean BMI values of 24.4 ± 7.5 kg/m^2^, 26.1 ± 8.3 kg/m^2^, and 25.8 ± 7.9 kg/m^2^, respectively (Table 1). The *p*-value of 0.749 suggests that there was no statistically significant difference in the average BMI across the groups.

The gender distribution among groups was also evaluated, with 50.0% of the mild group being male compared with 63.6% in the moderate group and 59.1% in the severe group. This gender distribution did not significantly differ among the groups (*p*-value = 0.647). Furthermore, the place of residence (urban vs. non-urban), the smoking status (current/former vs. non-smoker), and the Charlson Comorbidity Index (CCI ≥ 2, excluding lung disease) were also compared among the groups. However, none of these variables showed a significant difference across the severity groups, with *p*-values of 0.653, 0.419, and 0.370, respectively. The distribution of these variables suggests comparable baselines for the participants across different severity groups.

### 3.2. Pulmonary Function Tests

The Forced Vital Capacity (FVC) indicated a decrease with the severity of the disease. Patients with mild symptoms showed a mean FVC of 106.0 ± 18.9, while those with moderate and severe symptoms had lower average FVC values of 93.8 ± 21.2 and 86.8 ± 25.5, respectively, as described in Table 2. The decrease between the mild and severe groups was statistically significant (*p*-value = 0.018) as per the Tukey HSD Post Hoc Test. The difference in FVC values was approximately −19.2 (95% CI: −35.1 to −3.22, *p* = 0.014), indicating a significant reduction in lung function in patients with severe COVID-19 compared with those with mild symptoms.

Forced Expiratory Flow 25–75% (FEF25–75), which measures the flow of air coming out of the lungs during the middle portion of a forced exhale, was also found to decrease with the severity of COVID-19. The mild group had a mean value of 82.9 ± 5.6, while the moderate and severe groups had values of 79.2 ± 6.4 and 77.7 ± 6.0, respectively. The decrease between the mild and severe groups was statistically significant with a *p*-value of 0.017. Specifically, the Tukey HSD Post Hoc Test indicated a difference of −5.2 between the two groups (95% CI: −9.54 to −0.85, *p* = 0.0152), suggesting that the middle portion of the exhalation was significantly more impaired in the severe group as compared with the mild group.

However, no significant differences were observed in the Forced Expiratory Volume in 1 s (FEV1), the ratio of FEV1/FVC, and the Airway Microvascular Permeability (AMPI) among the three groups (*p*-values were 0.248, 0.319, and 0.249, respectively). When looking at the clinical outcomes, the number of patients with ventilatory dysfunction and post-COVID-19 fibrosis increased with disease severity (*p*-values were 0.049 and 0.021, respectively). The proportion of patients with a normal spirometry outcome decreased with increasing severity (*p* = 0.049).

Total Lung Capacity (TLC) showed a decreasing trend with the severity of the disease, with the mild group exhibiting a mean value of 119.1 ± 29.0, whereas the moderate and severe groups demonstrated lower values of 109.4 ± 40.6 and 100.8 ± 44.6, respectively. However, these differences were not statistically significant (*p*-value = 0.297), suggesting comparable TLC across the severity groups. Similarly, Residual Volume (RV) showed a decreasing pattern with increasing severity, but without a statistically significant difference (*p*-value = 0.465), as presented in Table 3.

In contrast, Specific Airway Resistance (sRAW) and the ratio of Residual Volume to Total Lung Capacity (RV/TLC) showed significant differences between the mild and severe groups in post hoc analysis. The mild group exhibited a mean sRAW of 58.2 ± 38.0 while the severe group had a lower average of 40.5 ± 21.2. The Tukey HSD Post Hoc Test indicated a significant difference of 106.4 (95% CI: 26.7 to 186.0, *p* = 0.005), revealing a significant increase in airway resistance in severe cases compared with mild ones.

The RV/TLC ratio was also different between the mild and severe groups, with the mild group having a lower mean value (35.0 ± 8.1) compared with the severe group (41.5 ± 9.6). The Tukey HSD Post Hoc Test showed a significant difference of 6.5 (95% CI: 0.20 to 12.7, *p* = 0.041), indicating a significant increase in the proportion of Residual Volume relative to Total Lung Capacity in severe cases. The other measurements, including Airway Resistance (RAW) and Airway Conductance (GAW), did not demonstrate statistically significant differences across the three groups. In terms of clinical outcomes, the number of patients with ventilatory dysfunction and post-COVID-19 fibrosis increased with the severity of COVID-19 (*p*-values were 0.009 and 0.038, respectively). The number of patients who were classified as ‘normal’ decreased with increasing severity (*p* = 0.017).

Table 4 of the study “Post-Infection Oscillometry and Pulmonary Metrics in SARS-CoV-2 Patients: A 40-Day Follow-Up Study” presents the oscillometry measurements of the participants at 40 days post-COVID-19 infection, categorized into mild, moderate, and severe groups. The Resonant Frequency (RF) showed an increasing trend with disease severity. The mean RF was 14.3 ± 4.5 for the mild group, 15.6 ± 2.9 for the moderate group, and 17.4 ± 4.4 for the severe group. This observed trend reached statistical significance (*p* = 0.042). The Tukey HSD Post Hoc Test further identified a significant difference in RF between the mild and severe groups with a mean difference of 3.1 (95% CI: 0.20 to 5.99, *p* = 0.033), suggesting a significant increase in RF in severe cases compared to mild cases.

In the case of Resistance at 4 Hz (R4), there was a decrease from the mild group (4.6 ± 2.1) to the severe group (3.5 ± 1.1). However, these differences did not reach statistical significance on post hoc analysis, even though the overall *p*-value was 0.035. Reactance at 4 Hz (X4) showed significant variation across the three groups with the mild group showing a mean of −2.2 ± 1.4, the moderate group showing −1.8 ± 1.1, and the severe group showing −2.8 ± 1.4. The Tukey HSD Post Hoc Test revealed a significant difference specifically between the moderate and severe groups with a mean difference of 1.0 (95% CI: 0.05 to 1.94, *p* = 0.036), implying increased negative reactance in severe cases compared to moderate cases. There were no statistically significant differences in Resistance at 6 Hz (R6), Reactance at 6 Hz (X6), and Resistance at 20 Hz (R20) among the three groups. 

The percentage of patients with ventilatory dysfunction was significantly higher in the severe group (72.7%) compared to the mild (31.8%) and moderate (45.5%) groups (*p* = 0.021). Meanwhile, the percentage of patients identified with post-COVID-19 fibrosis increased with disease severity, but the difference was not statistically significant (*p* = 0.310). The number of patients considered ‘normal’ was significantly reduced with increasing disease severity (*p* = 0.034).

Table 5 details the computed tomography findings of patients at 40 days post-COVID-19 infection, categorized into mild, moderate, and severe groups. The total lung volume, measured in liters, was comparable across all three groups (Mild: 4.3 ± 1.3, Moderate: 4.0 ± 0.9, Severe: 4.1 ± 1.1), with no statistically significant difference noted (*p* = 0.662). The left lung volume also demonstrated no significant variation across the groups (Mild: 1.9 ± 0.6, Moderate: 1.6 ± 0.8, Severe: 1.4 ± 0.9), with a *p*-value of 0.108. Similarly, the right lung volume did not exhibit a statistically significant difference among the groups (Mild: 2.1 ± 0.8, Moderate: 2.4 ± 1.0, Severe: 2.0 ± 1.2), with a *p*-value of 0.325.

The presence of emphysema was observed in 22.7%, 27.3%, and 40.9% of the mild, moderate, and severe groups, respectively, but this increase with disease severity did not reach statistical significance (*p* = 0.393). The occurrence of ground-glass opacities showed a significant increase with disease severity (*p* = 0.037), being present in 45.5% of the mild group, 54.5% of the moderate group, and 81.8% of the severe group. This suggests that ground-glass opacities were significantly more common in severe cases of COVID-19 at the 40-day mark. Crazy-paving patterns and consolidation were observed more frequently in the severe group than in the mild or moderate groups, but these differences did not reach statistical significance with *p*-values of 0.162 and 0.157, respectively.

The presence of vascular calcifications was noted only in the moderate (9.1%) and severe (13.6%) groups but was absent in the mild group. However, this difference was not statistically significant (*p* = 0.219). The number of patients with ‘normal’ results, on either the right or left lung or both, decreased with increasing disease severity, with 40.9% of mild cases, 18.2% of moderate cases, and 9.1% of severe cases being categorized as ‘normal’. This difference was statistically significant (*p* = 0.034).

### 3.3. Regression Analysis

X4 (Reactance at 4 Hz), FEF25–75 (Forced Expiratory Flow 25–75%), and RF (Resonant Frequency) showed significant relationships with the outcome of interest, as indicated by *p*-values of less than 0.05. Specifically, for every unit increase in X4, FEF25–75, and RF, the hazard, or the risk of the event occurring, was estimated to increase by a factor of 3.16 (95% CI: 2.15–7.39), 2.09 (95% CI: 1.48–2.70), and 1.90 (95% CI: 1.31–4.26), respectively.

**Figure 1:**
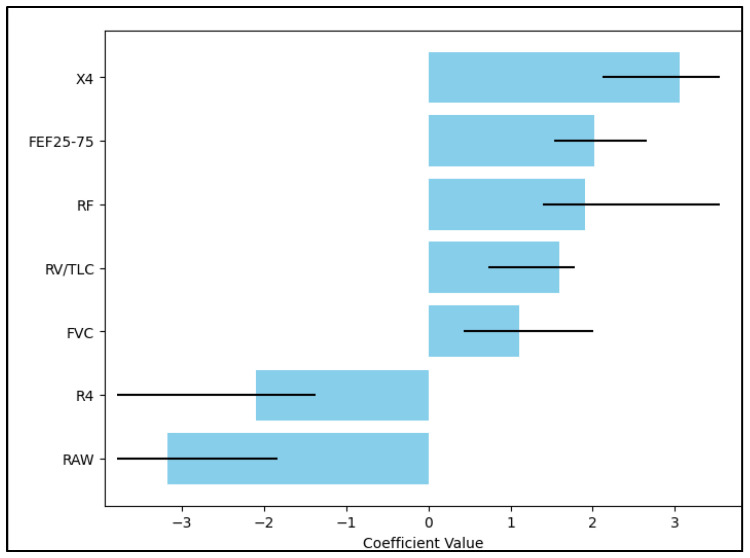
Multivariate regression.

In contrast, RV/TLC (Residual Volume to Total Lung Capacity ratio) and FVC (Forced Vital Capacity) did not significantly affect the hazard, with respective *p*-values of 0.219 and 0.440, indicating a lack of statistical significance. Interestingly, R4 (Resistance at 4 Hz) and RAW (Airway Resistance) were associated with decreased hazards, as indicated by negative Hazard Ratios (HRs) of −2.18 and −3.27, respectively. This means that, for every unit increase in R4 and RAW, the hazard decreased by a factor of 2.18 and 3.27, respectively. This relationship was statistically significant, with *p*-values of less than 0.001, as described in Table 6 and Figure 1 below.

## 4. Discussion

### 4.1. Literature Findings

The current study aimed to comprehensively evaluate pulmonary function and structure in patients with acute SARS-CoV-2 infection at a 40-day post-infection point. The results from our study revealed a number of interesting findings in spirometric parameters, plethysmography, forced oscillometry, and CT assessments. Our cohort of 66 participants, evenly divided into mild, moderate, and severe disease categories, showed no significant differences in terms of demographics, BMI, place of residence, or comorbidity index. This aligns with results of similar studies, such as the one conducted by Sonnweber et al. [18], thereby reinforcing that our findings are based on comparable baselines across the different severity groups.

Consistent with Mo et al. [19], our study identified a statistically significant reduction in Forced Vital Capacity (FVC) and Forced Expiratory Flow 25–75% (FEF25–75) in patients with severe COVID-19 compared with those with mild symptoms. These results further emphasize the deleterious effects of COVID-19 on lung capacity and the ability to forcibly exhale, especially in severe cases. Interestingly, we did not observe any significant differences in Forced Expiratory Volume in 1 s (FEV1), FEV1/FVC ratio, and Airway Microvascular Permeability (AMPI) across the severity groups. These findings are somewhat inconsistent with those of Mo et al., who reported a significant decline in FEV1 in severe cases. However, the discrepancy could be attributed to differences in the post-infection timeline of assessment.

Our study revealed that Total Lung Capacity (TLC) and Residual Volume (RV) showed a decreasing trend with disease severity, but the differences were not statistically significant, suggesting that these parameters are not significantly impacted by the disease severity at the 40-day mark. On the contrary, we found a significant increase in Specific Airway Resistance (sRAW) and the RV/TLC ratio in severe cases compared with mild ones. This observation aligns with the findings of Zhao et al. [20] who reported increased Airway Resistance and RV/TLC ratio in COVID-19 patients, indicating that severe SARS-CoV-2 infection is associated with more residual air and greater airway resistance.

The current study provides comprehensive data on the role of forced oscillometry in evaluating post-COVID-19 lung function. We found a significant increase in Resonant Frequency (RF) in severe cases compared with mild ones. Reactance at 4 Hz (X4) also showed significant variation across the severity groups, with increased negative reactance in severe cases compared with moderate ones. These findings add to the growing evidence that forced oscillometry could be an effective tool in identifying and quantifying subtle changes in lung function not detected using conventional pulmonary function tests.

We found no significant differences in total lung volume across severity groups in our CT assessments. These findings are similar to those of Han et al. [21] who reported comparable total lung volumes in their study of post-acute COVID-19 patients. However, our study found a significantly higher prevalence of ground-glass opacities in severe cases at the 40-day mark, in line with findings by Pan et al. [22]. This suggests that ground-glass opacities could be a persistent feature in patients with severe COVID-19, even after the acute phase of the disease.

Diving deeper into the available literature on post-infection oscillometry and pulmonary metrics in SARS-CoV-2 patients, several studies point towards the potential of oscillometry as a more sensitive measure of lung function impairment compared with traditional spirometry. A recent study by Pakhale et al. [23] demonstrated the clinical utility of oscillometry in identifying persistent lung function abnormalities in patients with mild to moderate COVID-19 disease and who had normal spirometric results. Furthermore, according to a study by Frija-Masson et al. [24], lung diffusion capacity, measured through single-breath diffusing capacity for carbon monoxide (DLCO), can also be impaired in COVID-19 patients, including those with mild disease who did not require hospitalization. Therefore, it is crucial to consider these functional abnormalities when following up with COVID-19 patients, irrespective of the disease severity during the acute phase.

Nevertheless, in the current study, the Cox regression model indicated that X4, FEF25–75, and RF were associated with an increased risk of the outcome, while R4 and RAW were associated with a decreased risk. RV/TLC and FVC did not significantly affect the outcome. It is important to note that these associations do not imply causality, and the interpretation of these variables should consider the clinical context and other potential confounding factors.

We also compared spirometry, body plethysmography, oscillometry, and CT scans to determine their accuracy in identifying ventilatory dysfunction and post-COVID-19 fibrosis. It was observed that the prevalence of ventilatory dysfunction and post-COVID-19 fibrosis increased with disease severity across all three measurement methods. However, there were no statistically significant differences between spirometry, body plethysmography, and oscillometry in detecting ventilatory dysfunction when compared with the CT findings. In terms of ventilatory dysfunction, the highest prevalence was observed in the severe group for spirometry (59.1%), body plethysmography (81.8%), and oscillometry (72.7%). Similarly, post-COVID-19 fibrosis showed an increasing trend with disease severity, with the severe group having the highest prevalence for all three measurement methods: spirometry (50.0%), body plethysmography (54.5%), and oscillometry (68.2%).

When comparing the methods with the CT findings, spirometry, body plethysmography, and oscillometry demonstrated comparable accuracy in identifying ventilatory dysfunction and post-COVID-19 fibrosis. Overall, our findings suggest that spirometry, body plethysmography, and oscillometry are effective methods for evaluating ventilatory dysfunction and post-COVID-19 fibrosis. These non-invasive and accessible techniques can provide valuable insights into the respiratory health of patients recovering from SARS-CoV-2 infection, offering viable alternatives to CT scans for routine assessments. Further research is warranted to optimize the integration of these methods and investigate their long-term correlation with clinical outcomes.

Nevertheless, it is assumed that vaccination can influence the short-term and long-term outcomes after COVID-19, as patients included in the current study were unvaccinated. The prevention and management of COVID-19 in long-term care facilities (LTCFs) and nursing homes are central themes across the provided abstracts. Infection control measures are emphasized, with strong evidence advocating for comprehensive interventions such as vaccination against influenza, SARS-CoV-2, and pneumococcal disease, as well as the implementation of robust infection control protocols including hand washing, social distancing, and the use of PPE [25,26]. Further studies have delved into the specific factors contributing to outbreaks, including facility size, staffing practices, and the unique environmental characteristics of nursing homes, suggesting the implementation of specific interventions like mass testing, visitor restrictions, and droplet/contact precautions to reduce transmission rates [26,27].

On the other hand, vaccinations and their effects are a focal point, with evidence demonstrating that the availability of influenza and pneumococcal vaccines has a positive impact on COVID-19 progression in the elderly population living in nursing homes [28]. This notion is supported by consensus statements that promote extensive influenza and pneumococcal vaccination campaigns, even hinting at possible protection against COVID-19 [29]. Epidemiology, morbidity, and mortality are also key concerns, with data provided on specific COVID-19 infection rates, outbreaks, and mortality. The effects of gender, chronic illness, and vaccination on COVID-19-related hospitalization and death are detailed, with male sex identified as a particular risk factor [29,30]. Importantly, the need to balance infection prevention with residents’ quality of life is a recurring theme, emphasizing the complexities of managing COVID-19 in these sensitive environments [27].

### 4.2. Study Limitations

The present study has several limitations that should be acknowledged. Firstly, the observational study design limits the ability to establish causal relationships. Secondly, the study was conducted at a single center, potentially limiting the generalizability of the findings. The small sample size and exclusion of individuals with pre-existing pulmonary diseases further restrict the generalizability of the results. The 40-day follow-up period may not capture long-term effects and recovery trajectories. The use of multiple assessment modalities introduces potential sources of error and limitations inherent to each method. Despite these limitations, this study provides important insights into post-SARS-CoV-2 infection pulmonary function and structure, which can inform future research and clinical management.

## 5. Conclusions

In conclusion, this study provided a comprehensive evaluation of pulmonary function and structure in patients with acute SARS-CoV-2 infection at 40 days post-infection. The findings revealed significant reductions in Forced Vital Capacity and Forced Expiratory Flow 25–75% with increasing disease severity. Total lung capacity showed a decreasing trend, although this was not statistically significant. Specific Airway Resistance and the RV/TLC ratio were significantly higher in severe cases. Oscillometry measurements demonstrated an increase in Resonant Frequency (RF) and negative reactance (X4) in severe cases. The computed tomography findings showed a higher prevalence of ground-glass opacities in severe cases. Regression analysis revealed significant relationships between X4, FEF25–75, RF, and the outcome of interest. Nevertheless, these findings contribute to the understanding of post-SARS-CoV-2 pulmonary function and provide perspectives for future research and clinical management.

## Figures and Tables

**Table 1:** Background of the COVID-19 patients.

Variables (Mean ± SD)	Mild (*n* = 22)	Moderate (*n* = 22)	Severe (*n* = 22)	*p*-Value
Age, years (mean ± SD)	54.1 ± 9.8	57.2 ± 8.6	58.3 ± 11.0	0.347
Sex, male (n, %)	11 (50.0%)	14 (63.6%)	13 (59.1%)	0.647
Place of residence, urban (n, %)	9 (40.9%)	12 (54.5%)	10 (45.5%)	0.653
BMI, kg/m^2^ (mean ± SD)	24.4 ± 7.5	26.1 ± 8.3	25.8 ± 7.9	0.749
Smoking, current/former (n, %)	7 (31.8%)	6 (27.3%)	10 (45.5%)	0.419
CCI ≥ 2 (n, %) *	8 (36.4%)	8 (36.4%)	12 (54.5%)	0.370

*—Excluding lung disease; SD—Standard Deviation; BMI—Body Mass Index; CCI—Charlson Comorbidity Index.

**Table 2:** Spirometry measurements at 40 days post-COVID-19.

Variables (Mean ± SD)	Mild (*n* = 22)	Moderate (*n* = 22)	Severe (*n* = 22)	*p*-Value
FVC	106.0 ± 18.9	93.8 ± 21.2	86.8 ± 25.5	0.018 *
FEV1	98.7 ± 22.0	92.6 ± 20.3	87.4 ± 24.2	0.248
FEV1/FVC	80.6 ± 7.3	82.5 ± 5.0	84.0 ± 9.3	0.319
FEF25–75	82.9 ± 5.6	79.2 ± 6.4	77.7 ± 6.0	0.017 *
AMPI	85.9 ± 14.3	90.6 ± 11.9	92.4 ± 13.3	0.249
Result (n, %)				
Ventilatory dysfunction	5 (22.7%)	9 (40.9%)	13 (59.1%)	0.049
Post-COVID-19 fibrosis	3 (13.6%)	5 (22.7%)	11 (50.0%)	0.021
Normal	14 (63.6%)	9 (40.9%)	6 (27.3%)	0.049

*—Significant on post hoc analysis; SD—Standard Deviation; FVC (Forced Vital Capacity); FEV1 (Forced Expiratory Volume in 1 s); FEF25–75 (Forced Expiratory Flow 25–75%); AMPI—Airway Microvascular Permeability.

**Table 3:** Body plethysmography measurements at 40 days post-COVID-19.

Variables (Mean ± SD)	Mild (*n* = 22)	Moderate (*n* = 22)	Severe (*n* = 22)	*p*-Value
TLC	119.1 ± 29.0	109.4 ± 40.6	100.8 ± 44.6	0.297
RV	204.7 ± 88.9	186.4 ± 82.2	171.7 ± 92.8	0.465
sRAW	58.2 ± 38.0	54.2 ± 24.2	40.5 ± 21.2	0.109
RAW	77.4 ± 40.0	108.6 ± 80.0	183.8 ± 168.2	0.007 *
GAW	185.6 ± 150.2	125.3 ± 129.4	113.9 ± 126.5	0.178
RV/TLC (%)	35.0 ± 8.1	39.3 ± 8.3	41.5 ± 9.6	0.048 *
Result (n, %)				
Ventilatory dysfunction	8 (36.4%)	13 (59.1%)	18 (81.8%)	0.009
Post-COVID-19 fibrosis	4 (18.2%)	7 (31.8%)	12 (54.5%)	0.038
Normal	12 (54.5%)	8 (36.4%)	3 (13.6%)	0.017

*—Significant on post hoc analysis; SD—Standard Deviation; TLC—Total Lung Capacity; RV—Residual Volume; sRAW—Specific Airway Resistance; RAW—Airway Resistance; GAW—Airway Conductance.

**Table 4:** Oscillometry measurements at 40 days post-COVID-19.

Variables (Mean ± SD)	Mild (*n* = 22)	Moderate (*n* = 22)	Severe (*n* = 22)	*p*-Value
RF	14.3 ± 4.5	15.6 ± 2.9	17.4 ± 4.4	0.042 *
R4	4.6 ± 2.1	4.6 ± 1.4	3.5 ± 1.1	0.035
R6	3.5 ± 1.6	3.8 ± 0.8	4.0 ± 1.0	0.375
X4	−2.2 ± 1.4	−1.8 ± 1.1	−2.8 ± 1.4	0.045 *
X6	−1.5 ± 0.7	−1.3 ± 0.5	−1.2 ± 0.5	0.219
R20	3.6 ± 1.5	3.0 ± 0.9	3.4 ± 1.5	0.320
Result (n, %)				
Ventilatory dysfunction	7 (31.8%)	10 (45.5%)	16 (72.7%)	0.021
Post-COVID-19 fibrosis	10 (45.5%)	12 (54.5%)	15 (68.2%)	0.310
Normal	9 (40.9%)	4 (18.2%)	2 (9.1%)	0.034

*—Significant on post hoc analysis; SD—Standard Deviation; RF—Resonant Frequency; R4—Resistance at 4 Hz; R6—Resistance at 6 Hz; X4—Reactance at 4 Hz; X6—Reactance at 6 Hz; R20—Resistance at 20 Hz.

**Table 5:** Computed tomography findings at 40 days post-COVID-19.

Variables (Mean ± SD)	Mild (*n* = 22)	Moderate (*n* = 22)	Severe (*n* = 22)	*p*-Value
Total lung volume (liters)	4.3 ± 1.3	4.0 ± 0.9	4.1 ± 1.1	0.662
Left lung volume (liters)	1.9 ± 0.6	1.6 ± 0.8	1.4 ± 0.9	0.108
Right lung volume (liters)	2.1 ± 0.8	2.4 ± 1.0	2.0 ± 1.2	0.325
Emphysema	5 (22.7%)	6 (27.3%)	9 (40.9%)	0.393
Ground glass	10 (45.5%)	12 (54.5%)	18 (81.8%)	0.037
Crazy paving	2 (9.1%)	4 (18.2%)	7 (31.8%)	0.162
Consolidation	1 (4.5%)	2 (9.1%)	5 (22.7%)	0.157
Vascular calcifications	0 (0.0%)	2 (9.1%)	3 (13.6%)	0.219
Normal (R/L or both)	9 (40.9%)	4 (18.2%)	2 (9.1%)	0.034

SD—Standard Deviation.

**Table 6:** Cox regression model.

Independent Variables	HR—Exp(B)	95% CI	*p*-Value
X4	3.16	2.15–7.39	<0.001
FEF25–75	2.09	1.48–2.70	0.001
RF	1.90	1.31–4.26	0.036
RV/TLC	1.66	0.68–1.92	0.219
FVC	1.15	0.42–2.03	0.440
R4	−2.18	−1.59–(−4.80)	<0.001
RAW	−3.27	−1.93–(−6.16)	<0.001

HR—Hazard Ratio; CI—Confidence Interval; X4—Reactance at 4 Hz; FEF25–75 (Forced Expiratory Flow 25–75%); RF—Resonant Frequency; RV—Residual Volume; TLC—Total Lung Capacity; FVC—Forced Vital Capacity; R4—Reactance at 4 Hz; RAW—Airway Resistance.

## Data Availability

Data available on request.
